# Red Cross Uses of Aerial Transport

**Published:** 1913-10-04

**Authors:** 


### THE RED CROSS USES OF AERIAL TRANSPORT.

The article on "The Probable Uses of Aerial Transport to the Medical Profession" which appeared in our issue of August 23 received so many comments, both from members of the profession and from the lay press, that it is permissible to refer again to the adverse criticisms which were also elicited.

Some have brought forward in support of their objection the fact that the very identical aeroplane with which Colonel Donegan's experiments were conducted unfortunately came to grief. Others allude to Mr. Hawker's failure in the all round Britain race, and some object to Colonel Donegan's proposed means of transport of medical officers on the field, pointing out that the services of the Army surgeon are so valuable in maintaining the health of the troops that they should not be exposed to such a dangerous mode of transport. This, if we may say so, is a view hardly likely to appeal to a corps of officers who have often to endanger their lives in the execution of their duty.

War has its dangers for all, and the fact that regimental officers are nowadays instructed in the laws of sanitation renders the Army more independent of its Medical Service as regards the maintenance of health than heretofore. In the Continental press notices which we have at our disposal proposals in the article appear to be looked on in a favourable light, and the fact that Great Britain is the first nation to attempt a practical system in this direction is freely admitted. This certainly reflects credit on our Army Medical Organising Staff, who, in this respect, cannot be accused of being behind the times.

In the August issue of the *Royal Army Medical Corps Journal* there appeared an article on first-aid outfits for aeroplanes by Captain E. O. R. Lithgow, in which he says: "Aircrafts have now become so reliable and powerful that it is quite practicable to have machines specially fitted to carry medical and surgical equipment; these machines could search for wounded on the field and enable cases to be treated on the spot." Captain Lithgow goes on to say that the question of Red Cross aeroplanes is already receiving the attention of the Powers Signatory to the Geneva Convention and that these aeroplanes must of necessity be piloted by medical officers. We do not question the authority of the *Royal Army Medical Corps Journal* on such a subject, but we can hardly see the necessity for aeroplanes in the event of war being invariably piloted by medical officers. If medical officers are in future to drive aeroplanes they should have been asked similarly to drive ambulance wagons and all carts connected with the Medical Service. As was remarked in the article on "Aerial Transport" already referred to, there is no objection to a medical practitioner driving his own aeroplane if he so desires, as he now drives his own motor car, but it is not desirable that the medical expert should be anything but a passenger.

An editorial note on experiments made by Raymond, a member of the French Senate and a doctor of medicine, appears in the *Royal Army Medical Corps Journal* for September. As regards the utility of aeroplanes in looking for wounded on the battlefield he also says that the aeroplane could be used as a means of communication between the director of medical services in the field and his medical units and also for rapid conveyance of a medical officer to an outlying post. We note that Mr. Grahame-White has at present at Hendon a biplane capable of carrying no less than five passengers comfortably seated, and that it has made numerous flights without accident. Our contributor's suggestions are, therefore, supported by the fact of such a machine being in existence, so that as time advances there is certainly the probability of seeing ideas realised as regards the utility of aerial transport to the medical profession. Suggestions for its future development are in the nature of prophecy, but prophecy has been proved before now to be the truth of to-morrow.

